# Effect of Steel Plates on Estimation of the Compressive Strength of Concrete via Ultrasonic Testing

**DOI:** 10.3390/ma13040887

**Published:** 2020-02-17

**Authors:** Hong Chul Rhim, Dae You Kim, Chang Shik Cho, Do Hyun Kim

**Affiliations:** 1 Department of Architectural Engineering, Yonsei University, Seoul 03722, Korea; kdy19910@naver.com; 2 Korea Fire Safety Association, Cheongju-Si, Chungcheongbuk-Do, Seoul 28620, Korea; changshik00@naver.com; 3 Chang Min woo Structural Consultants, Gangnam-Gu, Seoul 06125, Korea; dhkim@minwoo21.com

**Keywords:** ultrasonic test, compressive strength, reinforcement, concrete, steel plate

## Abstract

The presence of embedded steel affects the estimates obtained for the compressive strength of concrete during ultrasonic testing, as it increases the ultrasonic wave velocity. Thus, if the presence of steel in concrete is inevitable, then a correction factor is required for an accurate estimation of the concrete strength. While previous studies focused on the effect of steel reinforcing bars on the speed of ultrasonic waves in concrete, this work expands on the significance of embedded steel from steel bars to include steel plates. The wave velocity was measured for varying dimensions of embedded steel plates from 15 mm to 150 mm using 54-kHz ultrasonic testing equipment. Through experiments, the effect of steel plates on the ultrasonic testing of concrete was quantified to derive proper correction factors. It was found that the thickness, depth, and height of the steel plates significantly affected the test results. These findings can be applied to ultrasonic testing to estimate the compressive strength of concrete consisting of a significant volume of steel, such as in steel-reinforced concrete structures.

## 1. Introduction

To determine the compressive strength of concrete, destructive coring tests as well as nondestructive testing (NDT) methods such as the rebound hammer, ultrasonic tests, and radar are widely used [1]. However, because each testing method has advantages and disadvantages, continuous efforts are still being made to improve the accuracy and reliability of these methods [2,3].

Test methods are also categorized into penetration and nonpenetration modes. Because concrete typically exhibits variations in its material property even within a single member, concrete strengths are better obtained via penetration mode tests such as in ultrasonic testing by passing through the entire cross-section of a member rather than part of it, as in the nonpenetration or reflection mode. However, ultrasonic waves are known to be affected by the presence of embedded steel inside the concrete. Thus, it is necessary to quantitatively investigate the significance of the effect of steel.

When dealing with the effect of steel in concrete on the estimation of the compressive strength of concrete, two scenarios need to be considered. First, it has been reported that when an ultrasonic wave crosses the steel bars in a direction that cuts through the cross-sectional areas, the summation of the cross-sectional areas of steel causes the wave velocity to increase [4,5]. Second, it has been reported that when the wave travels through the steel bars in a direction parallel to the length of the bars, the wave velocity also increases [5,6]. Based on these reports, the codes of some countries recommend that testing methods apply correction factors [7]. Furthermore, some countries do not allow ultrasonic testing when steel is present inside concrete [8].

Currently, steel-reinforced concrete (SRC) structures are used extensively worldwide. In SRC structures, in addition to traditional steel reinforcing bars, steel plates are incorporated to increase the load-carrying capacity [9,10]. Thus, in such cases, to accurately estimate the concrete strength, the effect of steel plates when ultrasonic waves are used for testing must be examined. Previous research works include the influence of steel reinforcement on ultrasonic velocity [11] and the use of ultrasonic testing to monitor the curing concrete [12,13].

In this study, the effect of steel plates on ultrasonic testing for estimating the compressive strength of concrete was quantified through a series of experiments. The results obtained indicate that the thickness, depth, and height(that is, the dimensions of the steel plates) affect the test results significantly. The findings can be applied when ultrasonic testing is utilized to estimate the compressive strength of concrete consisting of a significant volume of steel, such as in SRC structures. The work presented in this paper can be applied to estimate the compressive strength of concrete in the field when the exact inside layout of the section is known. 

## 2. Research Motivation

### 2.1. Concrete Strength Test in the Field

Unlike metal or metal-based materials, the testing of concrete has an intrinsic uncertainty owing to its highly heterogeneous nature. The proper estimation of concrete strength in the field during construction is always an important task to be performed. Usually, control cylinders are made on-site as ready-mixed concrete is delivered by trucks, and the cylinders are tested to ensure the strength of the concrete cast inside the formworks. However, the curing conditions of these cylinders and those of actual cast concrete as building members are different. Again, because of their heterogeneous nature, it is helpful to have some data from the cylinders, but the results cannot guarantee the quality of the actual cast concrete. Thus, it would be helpful if the curing conditions of cast concrete can be more accurately monitored in the field. 

In addition to the heterogeneous problem of concrete, reinforced or steel-reinforced concrete structures have steel reinforcing bars and/or steel plates. This further complicates the concrete strength estimation. The effect of steel on the wave velocity inside concrete has been reported in many previous studies; however, the effect of steel plates on the velocity inside an actual steel-reinforced concrete member has never been reported. With the increased use of steel plates inside concrete structures, it is now necessary to address this situation in terms of concrete strength tests in the field.

### 2.2. Field Situation

**Figure 1:**
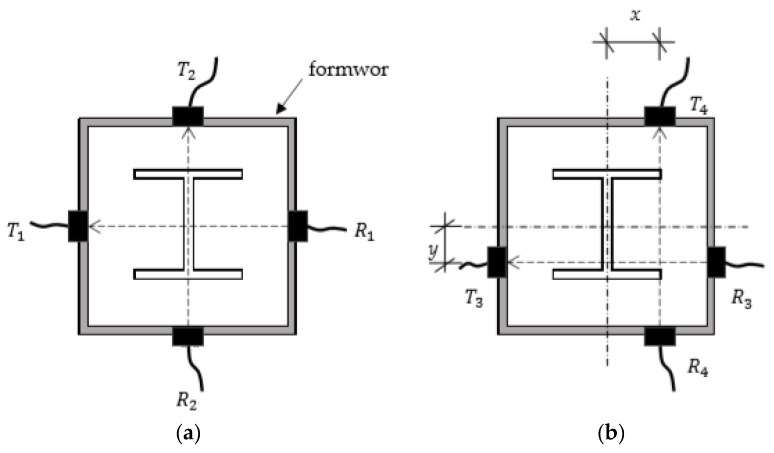
Combination of transmitter (T) and receiver (R) location (**a**) at center of section and (**b**) with offset from center.

In the field, a concrete member under testing is covered with a formwork, as shown in Figure 1. If there were no steel plates inside, the best location for placing probes was along the centerline, as shown in Figure 1a, because the effect of the edge is minimized along the centerline. However, the centerline might be blocked from measurements depending on the situation around the member in the field. It is also more effective to measure the wave velocity, avoiding the area of the steel plates, as shown in Figure 1b.

**Figure 2:**
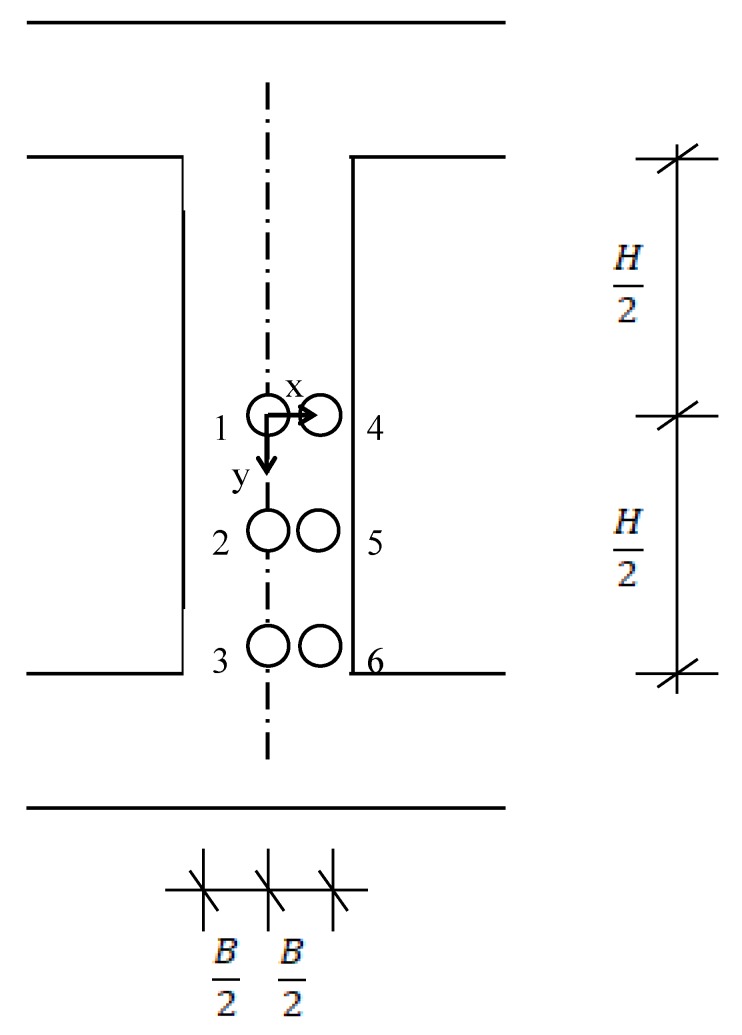
Possible locations along column to place a probe. H: height of column; B: side dimension of column.

A proper location for measurements can be identified, as shown in Figure 2. Once the location is determined, an opening to place probes can be created on the formwork. In Figure 2, an example of possible locations for placing probes is shown. As such, it is now easy to place probes at predesignated locations. 

### 2.3. Proposed Application Method

**Figure 3:**
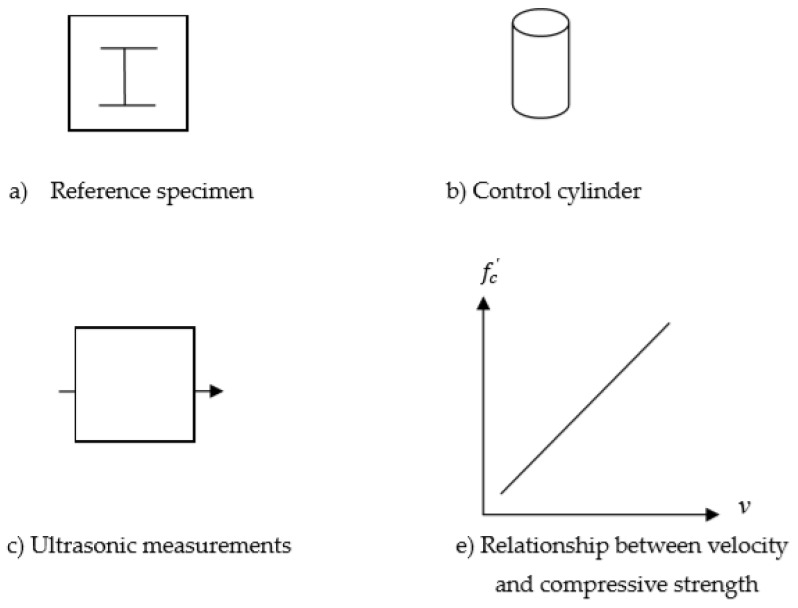
Procedures for field application.

To measure the compressive strength of the concrete of a steel-plate-embedded concrete member in the field, the following procedure is proposed, as shown in Figure 3. This method is proposed for freshly cast concrete under the condition that the inside layout of the steel plates is known.

(a) The reference specimen is made on-site with the same inside configuration as that to be built for a structure under construction.
(b) Control cylinders were made to measure the compressive strength of concrete over the curing time as often as needed.
(c) Ultrasonic measurements were conducted at a predesignated place for a given curing age. This was repeated for up to four weeks, which is considered when 100aconcrete strength of is achieved.
(d) At each ultrasonic measurement time in Step (c), the compressive strength of the control cylinders is measured.
(e) Finally, a database or graph is obtained for the ultrasonic velocity vs. the compressive strength of concrete.
(f) Now, it is time to measure the velocity of a real steel-plate-reinforced concrete member. Once the value is obtained, it is compared to the graph in Step (e) so that the compressive strength of the concrete in the member can be estimated.

## 3. Related Theory and Experimental Setup 

### 3.1. Ultrasonic Waves in Concrete for Compressive Strength Testing 

Ultrasonic waves are used during the NDT of concrete to determine the thickness of the concrete [14], the location of the steel reinforcing bars, and the presence of cracks [15]. Ultrasonic testing is also conducted in association with other NDT methodssuch as the rebound hammer method to improve the accuracy of the tests [16]. 

The velocity of an ultrasonic wave can be determined using Equation (1) [7]. As the strength of the concrete increases, the velocity also increases. The presence of steel inside the concrete further increases the velocity depending on its dimensions. Thus, the presence of steel reinforcing bars or plates can be ascertained by comparing the velocity of the wave to its velocity in plain concrete without any steel.

The pulse velocity V (m/s) of longitudinal stress waves in a concrete mass is related to its elastic properties and density according to the following relationship:

(1) $$V=\sqrt{\frac{E\left(1-\mu \right)}{\rho \left(1+\mu \right)\left(1-2\mu \right)}}$$

where E is the dynamic modulus of elasticity (MPa), µ is the dynamic Poisson’s ratio, and ρ is the density (kg/m^3^). In concrete, the elastic modulus of *E_c_* is directly related to the strength of the concrete, $f_{c}^{\prime }$. as suggested by the American Concrete Institute.

(2) $$E_{c}=33w_{c}^{1.5}\sqrt{f_{c}^{\prime }}$$

where $w_{c}$ is the weight of concrete (pounds per cubic foot), and $w_{c}^{1.5}$ is the compressive strength of concrete at 28 days in psi.

This study investigated how the volume of steel affects the wave velocity, with the objective of applying the quantitative results obtained to the ultrasonic NDT of concrete with steel plates inside. 

### 3.2. Specimens 

A mortar mix was used to avoid the wave propagation complexity that arises when a concrete mix with gravel is used. The mix ratio of the mortar was water:cement:sand at 1:2:4. Its28-day compressive strength was 19.85 MPa. 

### 3.3. Equipment

For the ultrasonic measurements, 54-kHz ultrasonic testing equipment(Pundit Lab Ultrasonic Instrument by Proceq in Switzerland) in the form of a transmitter and receiver was used. The probe was50 mm in diameter and 46 mm in height, and weighed287 g. The contact surface diameterwas36.77 mm. It was a single-crystal type with a bandwidth of less than 10 kHz. The piezoelectric electrical cell capacity was2 nF. The receiver gain was selected 100 times, and the excitation voltage was set to 125, 250, or 350 V. 

### 3.4. Test Specimens and Experimental Setup

**Figure 4:**
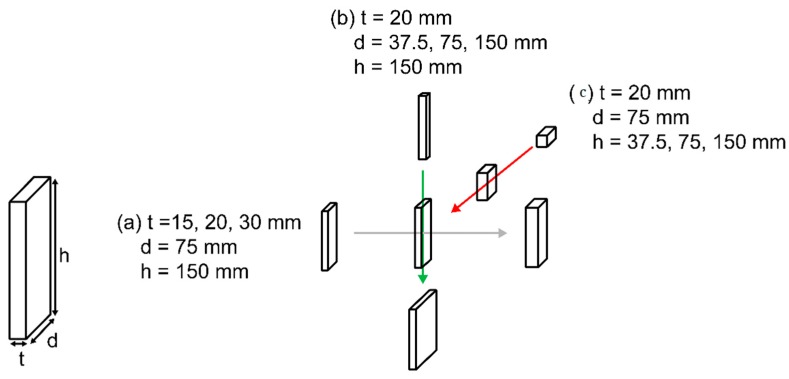
Measurement parameters:(**a**) thickness (t); (**b**) depth (d); (**c**) height (h) variation.

To examine the effect of steel plates on the velocity of ultrasonic waves in concrete, the parameters of thickness (t), depth (d), and height (h) of the steel plates were varied (Figure 4).

**Figure 5:**
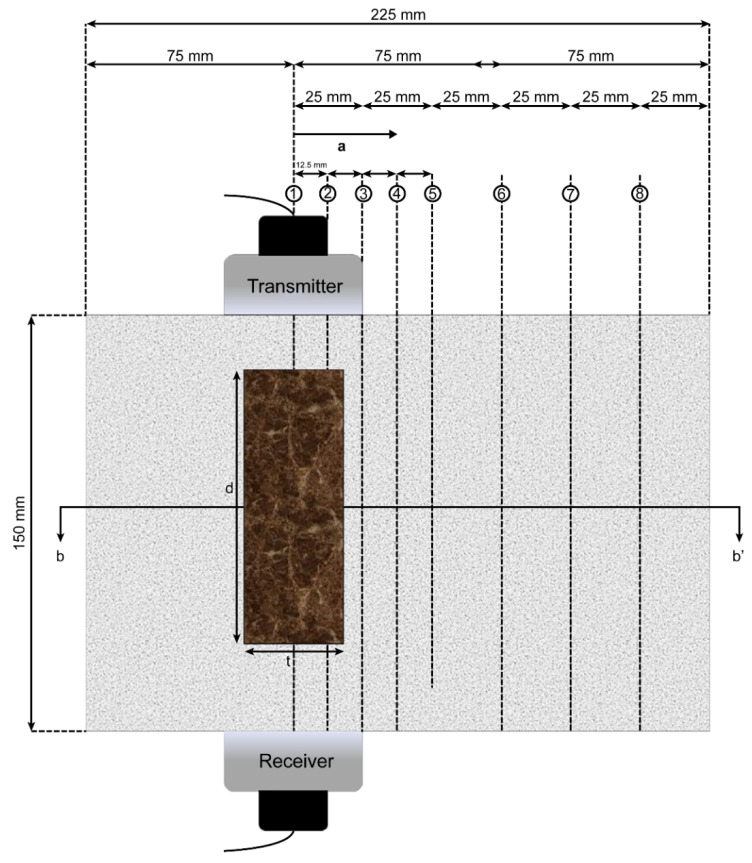
Top view of specimen with embedded steel plate.

**Figure 6:**
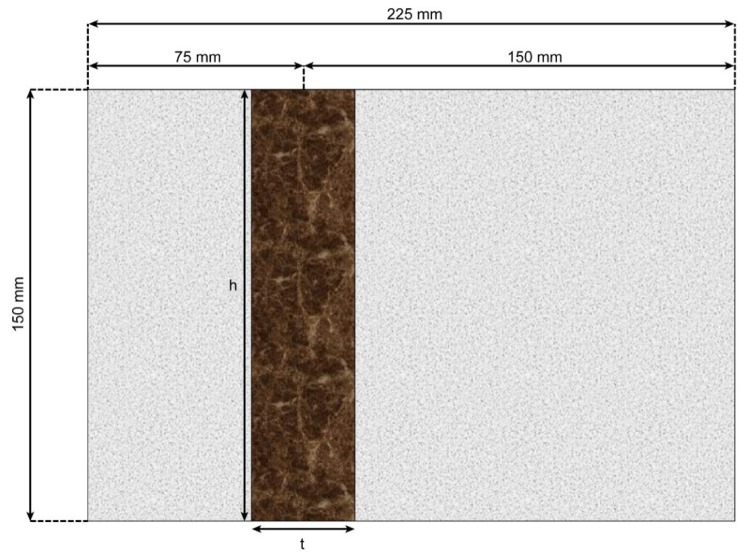
b-b’section of specimen.

Thus, seven steel plates of different dimensions were obtained. For each set of measurements, one parameter was varied, and the other two were kept constant. Each plate was placed inside a mortar specimen with dimensions of 150 mm × 150 mm × 225 mm (Figure 5 and Figure 6). 

The probes were placed along the depth of a plate at a distance *a* (mm) that was varied from the centerline of the plate, as shown in Figure 5. Eight locations were used for the measurements to determine the effect of the offset from the plate. A section view of the measurement setup is shown in Figure 6.

## 4. Results

### 4.1. Results Obtained by Varying Plate Thickness

The first set of measurements was made while varying the thickness of the plate. With the depth and height fixed at 75 mm and 150 mm, respectively, the thicknesses of the plates were varied, with values of 15 mm, 20 mm, and 30 mm, as shown schematically in Figure 4a. As the thickness of the plate increased, its effect on the wave velocity was examined. Four specimens were used for the measurements, including a specimen without a steel plate (as a reference). Eight ultrasonic measurements were made at the centerline of the plate and at certain distances away from the centerline (denoted as the variable distance a in Figure 5). 

**Figure 7:**
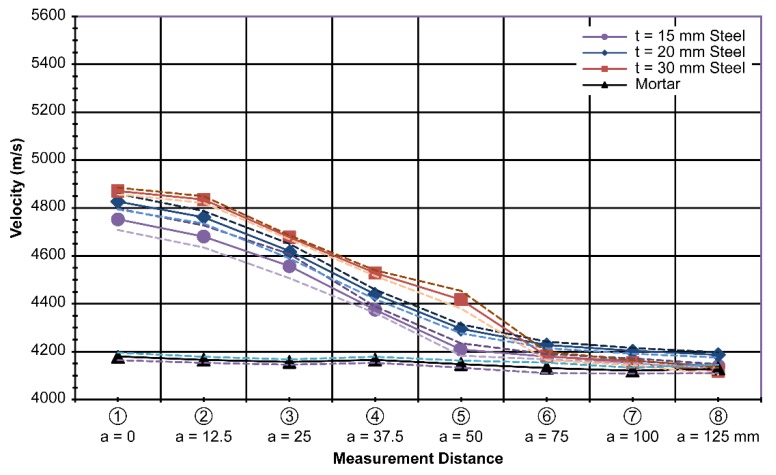
Measured velocity with varying thickness (t)at d = 75 mm and h = 150 mm.

For each measurement point, a total of 15 measurements were taken, and the average value and standard deviation of each measurement are shown in Figure 7. 

First, the velocity through a mortar specimen was 4150 m/s on average with a standard deviation of 25 m/s, which is 0.6% of the average value. This is regarded as quite stable, and is expected owing to the homogeneity of the mortar. Using this as a reference, the measured velocity with different thicknesses of the steel plates is plotted in Figure 7. 

In general, the specimens with the steel plates showed a significant difference in the measured velocity compared to the mortar. The 30-mm-thick plate specimen showed the highest velocity of 4871 m/s, followed by the 20-mm-thick plate specimen, which showed a velocity of 4827 m/s. Finally, the 15-mm-thick plate specimen showed a velocity of 4753 m/s along the centerline. The standard deviations for the 30-mm-, 20-mm-, and 15-mm-thick plate specimens were 14 m/s, 33 m/s, and 45 m/s, respectively. Therefore, the results were stable. 

Because the probes were placed farther away from the centerline of the steel plate, it is clear that the effect of the presence of the steel plate on the wave velocity diminished, as shown by the decreasing lines over the a axis in Figure 7. The difference in the measured velocity decreased beyond 75 mm along the a axis. Thus, it is suggested that, practically, ultrasonic probes must be placed exactly along the centerline of the steel plate or within a distance of 75 mm to establish the effect of the steel plate. 

**Figure 8:**
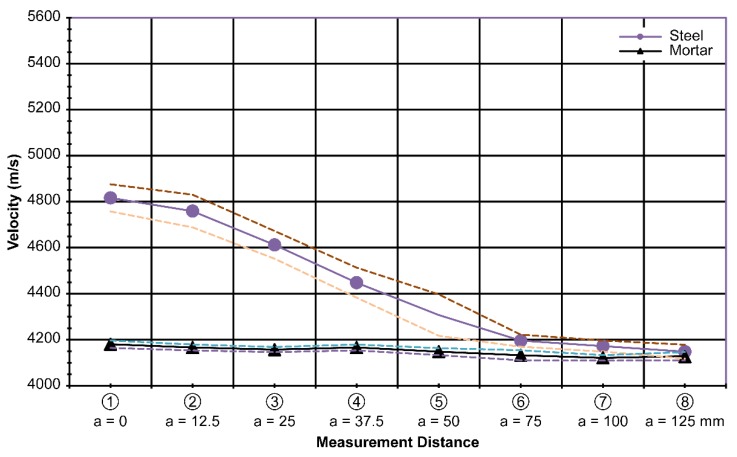
Combined measurement data with steel thickness (t)variation at d = 75 mm and h = 150 mm.

Even though the results with the steel plates showed a clear difference from the mortar results, the distinctions between different thicknesses overlapped somewhat, especially considering the band created by the standard deviation. The measurement results showed that it was quite possible to consider the effect of the steel plate when determining the compressive strength of concrete using ultrasonic testing, as shown in Figure 8. 

Figure 8 combines all of the measurement results for varying thicknesses(t). The standard deviation of the measurements with varying thicknesses of the steel plate was 204 m/s.

### 4.2. Results Obtained by Varying Plate Depth 

The second set of measurements was obtained while varying the depth of the plate inside the mortar specimen. Four specimens were used for the measurements. With the thickness and height fixed at 20 mm and 150 mm, respectively, the depth of the plates was varied over the values of 37.5, 75, and 150 mm, which corresponded to 25%, 50%, and 100% of the maximum depth, respectively. The depth denotes a direction parallel to the wave propagation. This depth change will significantly affect the wave velocity. Eight ultrasonic measurements were made at the centerline of the plate and at certain distances away from the centerline, which are denoted as a variable distance *a* in Figure 5. 

**Figure 9:**
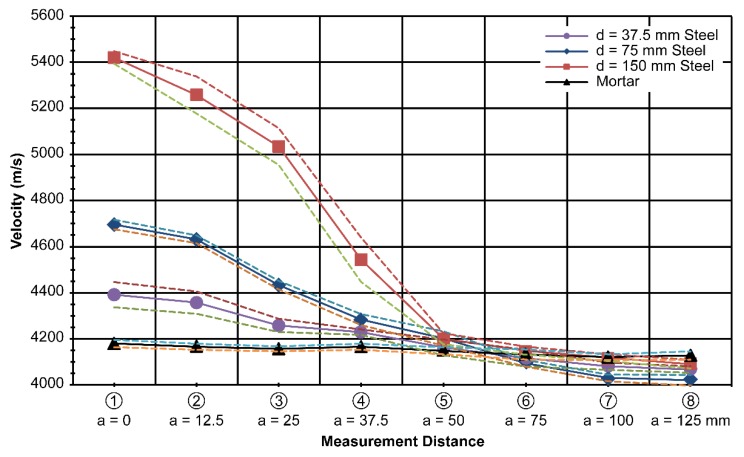
Measured velocity with varying depth (d)at t = 20 mm and h = 150 mm.

The results obtained showed a significant distinction in the measured velocity as the depth of the steel plate was varied, as shown in Figure 9. 

The average velocity of the wave in the mortar was 4180 m/s. It increased to 4392 m/s for the path along the 37.5-mm-deep steel plate, 4696 m/s for the path along the 75-mm-deep steel plate, and finally, 5421 m/s for the path along the 150-mm-deep steel plate. The wave velocity was significantly affected by the length of the steel located parallel to the direction of wave propagation. The results can be used as a basis for considering the effect of steel on the ultrasonic testing of reinforced concrete. The standard deviation for the 37.5-mm-deep plate specimen was 55 m/s; for the 75-mm-deep plate specimen,21 m/s; and for the 150-mm-deep plate specimen, 27 m/s, along the centerline of the plates. 

Similar to the thickness variation results in Figure 7 and Figure 8, the velocity difference diminished as the measurements were taken farther away from the centerline of the plate, especially after a distance of 50 mm, as shown in Figure 9. As the velocity difference is clearly seen in Figure 9, the results represent the purpose of this study well. By excluding the effect of steel, the physical properties of concrete in steel-reinforced concrete structures can be separated in terms of the ultrasonic wave velocity in relation to the compressive strength of concrete.

### 4.3. Results Obtained by Varying Plate Height 

The third set of measurements was made while varying the height of the plate. Four specimens were used for the measurements. With the thickness and depth of the steel plate fixed at 20 mm and 75 mm, respectively, the height of the plates was varied over the values of 37.5 mm, 75 mm, and 150 mm, which corresponded to 25%, 50%, and 100% of the maximum height, respectively. Eight ultrasonic measurements were made at the centerline of the plate and at several distances away from the centerline, denoted as variable distance *a* in Figure 5. 

In contrast to the previous set of measurements, only marginal differences were observed among the measurements. This implies that ultrasonic measurement is not very sensitive to height. However, the measured velocity was greater than that in concrete with no plate inside. 

**Figure 10:**
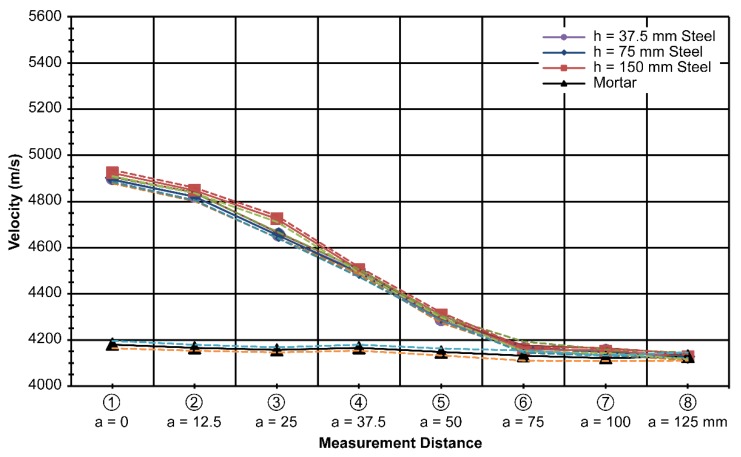
Measured velocity with varying height (h) at t = 20 mm and d = 75 mm.

**Figure 11:**
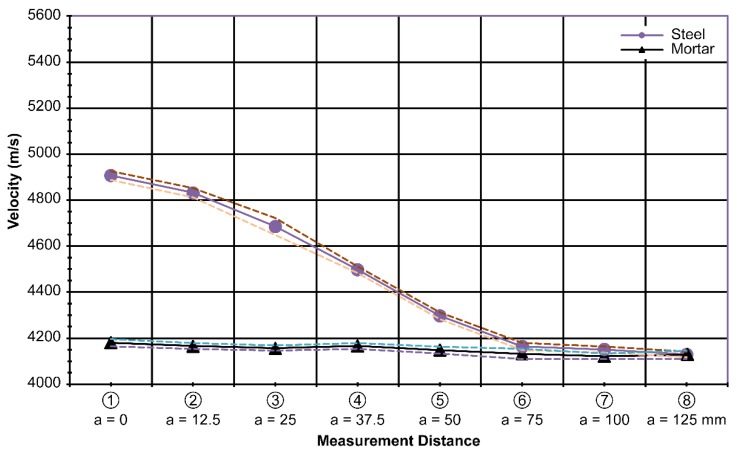
Integrated measurements according to varying steel heights(h)at t = 20 mm and d = 75 mm.

The average velocity of the wave for the 37.5-mm-high plate was 4893 m/s; for the 75-mm-high plate, 4895 m/s; and for the 150-mm-high plate,4924 m/s. The standard deviation for the 37.5-mm-high plate specimen was 15 m/s; for the 75-mm-high plate specimen, 10 m/s; and for the 150-mm-high plate specimen, 13 m/s, along the centerline of the plates. Similar to the previous results in Figure 7 and Figure 8, the velocity difference diminished as the measurements were taken farther away from the centerline of the plate, especially after a distance of 50 mm, as shown in Figure 10. The combined results of varying height h are shown in Figure 11.

The average standard deviation of the combined results with varying heightsof the steel plate was 18 m/s. It seems to be acceptable to use Figure 11 instead of Figure 10 because there is not much difference in the measured results of the height variation.

## 5. Discussion and Analysis

The purpose of conducting these experiments was to investigate the effect of steel on the measurement velocity of an ultrasonic wave so that the variation in wave velocity owing to different types of steel can be quantitatively studied. In practice, steel is inevitably present inside concrete. Ultrasonic test measurements are required to relate the measured velocity to the compressive strength of concrete, which is the most important property of concrete as it is being cured. 

The findings from the results of these measurements can provide useful relationships to determine the velocity of the wave in concrete with steel. This can be achieved by subtracting the value of the velocity owing to steel from the velocity measurement of the combined steel and concrete. Finally, the corresponding velocity of only concrete is obtained and is related to the compressive strength of concrete. The entire procedure is explained as follows:

In the laboratory, a database contains different steel shapes that are used practically and at different offset distances from the steel (as expected in the field). Then, we obtain the velocity difference data, as shown in Figure 7, Figure 8, Figure 9, Figure 10 and Figure 11.

(3) $$\Delta V=V_{\mathrm{stee}linconcrete}-V_{concreteonly}$$

From the laboratory measurements, the database for ΔV for three different measurement cases is listed in Table 1, Table 2 and Table 3.

Practically, for the actual application of this method, the ultrasonic velocity is measured with known steel shapes and at known offset distances from the steel $V_{\mathrm{stee}linconcrete}$. Then, from the premeasured database in the laboratory, which has measurement parameters identical to those in the field, the velocity in concrete only is obtained as in Equation (4).

(4) $$V_{\mathrm{steel}\;\mathrm{in}\;\mathrm{concrete}}-\Delta V_{fromdatabase}=V_{concreteonly}$$

**Figure 12:**
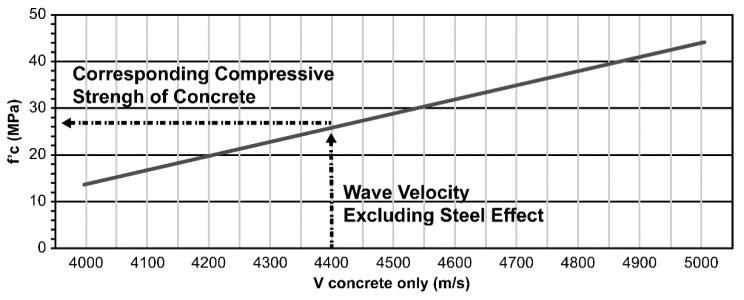
Velocity vs.compressive strength.

Finally, the compressive strength of concrete was evaluated from the premade velocity-vs.-strength graph. This graph can be easily obtained by making concrete specimens with different strengths and corresponding ultrasonic measurements. An example of such a graph is shown in Figure 12. 

## 6. Conclusions

The results of this study showed that the thickness, length, and height of the steel plates significantly affect the ultrasonic wave velocity, depending on the size and orientation of the plates. This paper proposed a method for determining the compressive strength of concrete in the field when there is a steel plate inside. By preestablishing a database in the laboratory with the same conditions of the steel layout as in the field, it is possible to exclude the effect of the increased portion of the velocity owing to the steel. The findings from this study can be applied when ultrasonic testing is utilized to estimate the compressive strength of concrete consisting of a significant volume of steel, such as in SRC structures. The work presented here applies to fresh concrete with an inside steel layout that is known beforehand. For already hardened and existing concrete members, a different approach needs to be studied. 

## Figures and Tables

**Table 1:** ΔV (m/s) for thickness variation at d = 75 mm and h = 150 mm.

Steel Thickness (mm)	Offset Distance *a* from Centerline of Steel (mm)				
	0	12.5	25	37.5	50
15	743 (100%)	670 (90%)	505 (68%)	346 (47%)	133 (18%)
20	745 (100%)	671 (90%)	506 (68%)	340 (46%)	145 (19%)
30	744 (100%)	698 (94%)	575 (77%)	354 (48%)	158 (21%)

**Table 2:** ΔV (m/s) for depth variation at t = 20 mm and h = 150 mm.

Steel Thickness (mm)	Offset Distance *a* from Centerline of Steel (mm)				
	0	12.5	25	37.5	50
37.5	242 (100%)	208 (86%)	108 (45%)	79 (33%)	13 (5%)
75	546 (100%)	482 (88%)	283 (52%)	113 (21%)	52 (10%)
150	1271 (100%)	1108 (87%)	884 (70%)	393 (31%)	51 (4%)

**Table 3:** ΔV (m/s) for height variation at t = 20 mm and d = 75 mm.

Steel Thickness (mm)	Offset Distance *a* from Centerline of Steel (mm)				
	0	12.5	25	37.5	50
All thicknesses combined	667 (100%)	609 (91%)	462 (69%)	297 (45%)	157 (24%)
